# Supplementary material for: Empirical methods for controlling false positives and estimating confidence in ChIP-Seq peaks
Source: BMC Bioinformatics. 2008 Dec 5;9:523. doi: 10.1186/1471-2105-9-523 (PMC2628906; doi:10.1186/1471-2105-9-523)
Supplement: Additional File 1 — A variety of html documents from the USeq web site detailing the available applications, their best usage, output file type descriptions, command line menus, etc. [file 1471-2105-9-523-S1.zip › USeqUserGuides/JavaDoc.zip!/JavaDoc/selex/UtilSelex.html]

UtilSelex


|  |  |  |
| --- | --- | --- |
| **Overview** \| **Package** \| **Class** \| **Use** \| **Tree** \| **Deprecated** \| **Index** \| **Help** | |  |
| **PREV CLASS**   NEXT CLASS | **FRAMES**    **NO FRAMES**     **All Classes** |  |
| SUMMARY: NESTED \| FIELD \| CONSTR \| METHOD | DETAIL: FIELD \| CONSTR \| METHOD |  |


---


## selex Class UtilSelex

```
java.lang.Object
  selex.UtilSelex
```

---

``` public class UtilSelex extends java.lang.Object ```

Utilites for the Selex package.

---

**Constructor Summary**

`UtilSelex()`


| **Method Summary** | |
| --- | --- |
| `static java.lang.String` | `averageDoubleArrayList(java.util.ArrayList Doubles)`             Averages an ArrayList of Double objects. |
| `static int` | `averageIntegerArrayList(java.util.ArrayList Integers)`             Averages an ArrayList of Integer objects. |
| `static void` | `printDoc()` |

**Methods inherited from class java.lang.Object**

`equals, getClass, hashCode, notify, notifyAll, toString, wait, wait, wait`

**Constructor Detail**

### UtilSelex

```
public UtilSelex()
```


**Method Detail**

### averageIntegerArrayList

```
public static int averageIntegerArrayList(java.util.ArrayList Integers)
```

:   Averages an ArrayList of Integer objects.

---


### averageDoubleArrayList

```
public static java.lang.String averageDoubleArrayList(java.util.ArrayList Doubles)
```

:   Averages an ArrayList of Double objects.

---


### printDoc

```
public static void printDoc()
```


---


|  |  |  |
| --- | --- | --- |
| **Overview** \| **Package** \| **Class** \| **Use** \| **Tree** \| **Deprecated** \| **Index** \| **Help** | |  |
| **PREV CLASS**   NEXT CLASS | **FRAMES**    **NO FRAMES**     **All Classes** |  |
| SUMMARY: NESTED \| FIELD \| CONSTR \| METHOD | DETAIL: FIELD \| CONSTR \| METHOD |  |


---
